# DeepBouton: Automated Identification of Single-Neuron Axonal Boutons at the Brain-Wide Scale

**DOI:** 10.3389/fninf.2019.00025

**Published:** 2019-04-18

**Authors:** Shenghua Cheng, Xiaojun Wang, Yurong Liu, Lei Su, Tingwei Quan, Ning Li, Fangfang Yin, Feng Xiong, Xiaomao Liu, Qingming Luo, Hui Gong, Shaoqun Zeng

**Affiliations:** ^1^Britton Chance Center for Biomedical Photonics, Wuhan National Laboratory for Optoelectronics-Huazhong University of Science and Technology, Wuhan, China; ^2^MoE Key Laboratory for Biomedical Photonics, Collaborative Innovation Center for Biomedical Engineering, School of Engineering Sciences, Huazhong University of Science and Technology, Wuhan, China; ^3^School of Mathematics and Statistics, Huazhong University of Science and Technology, Wuhan, China

**Keywords:** DeepBouton, single-neuron, axonal bouton, deep convolutional neural network, density-peak clustering

## Abstract

Fine morphological reconstruction of individual neurons across the entire brain is essential for mapping brain circuits. Inference of presynaptic axonal boutons, as a key part of single-neuron fine reconstruction, is critical for interpreting the patterns of neural circuit wiring schemes. However, automated bouton identification remains challenging for current neuron reconstruction tools, as they focus mainly on neurite skeleton drawing and have difficulties accurately quantifying bouton morphology. Here, we developed an automated method for recognizing single-neuron axonal boutons in whole-brain fluorescence microscopy datasets. The method is based on deep convolutional neural networks and density-peak clustering. High-dimensional feature representations of bouton morphology can be learned adaptively through convolutional networks and used for bouton recognition and subtype classification. We demonstrate that the approach is effective for detecting single-neuron boutons at the brain-wide scale for both long-range pyramidal projection neurons and local interneurons.

## Introduction

Mapping neural circuits, a core goal of modern neuroscience, depends on fine morphological reconstruction of individual neurons across the whole brain, including neuronal skeleton drawing and synaptic connectivity inference (Halavi et al., 2012; Helmstaedter and Mitra, 2012). Axonal boutons in optical microscopy images are typical presynaptic structures indicative of one or more synapses (Hellwig et al., 1994; Anderson et al., 1998). Recent research by Gala et al. (2017) and Drawitsch et al. (2018) showed that optical microscopy-based axonal boutons were highly correlative with electron microscopy. Therefore, identification of axonal boutons of individual neurons is critical for interpreting the patterns of neural circuit wiring schemes, as boutons indicate contact sites of individual neurons and reveal how neural circuits are wired (Braitenberg and Schüz, 1998; Lichtman and Denk, 2011). Furthermore, acquired bouton distribution patterns at the single-neuron level provide more comprehensive and finer structural information for defining cell types (Karube et al., 2004; Portera-Cailliau et al., 2005; Huang, 2014) and simulating neural circuits (Goodman and Brette, 2008; Brüderle et al., 2009; Markram et al., 2015) combined with neuronal arborization patterns.

Recent progress in fluorescence sparse-labeling and large-volume fine-imaging techniques (Micheva and Smith, 2007; Rotolo et al., 2008; Osten and Margrie, 2013; Economo et al., 2016; Gong et al., 2016) has enabled the acquisition of submicron-resolution whole-brain datasets of neuronal morphology. These techniques provide detailed structural information on single neuron and axonal boutons. Manual counting of axonal boutons in whole-brain datasets is extremely inconvenient and time-consuming given the large number and wide range of single-neuron boutons. As such, various algorithms and tools have been developed for automated reconstruction of individual neurons (Donohue and Ascoli, 2011; Myatt et al., 2012; Peng et al., 2015). Most of these approaches are able to extract neuronal skeletons well. However, these tools focus mainly on neurite tracing and are insufficient to precisely quantify bouton morphology.

Several methods for detecting axonal boutons from light microscopy images have been proposed. Song et al. (2016) proposed a score index for quantifying axonal boutons, which used the maximum intensity along the axon to locate boutons. Bass et al. (2017) developed an automated algorithm for detecting axonal boutons based on Gabor filters and support vector machine in local image volume. The primary principle underlying these approaches is using manually designed features to approximately model axonal boutons. Nevertheless, the features are insufficient to accurately describe complex bouton morphology, since there are many suspected axonal swellings similar to boutons derived from the inhomogeneities of axonal fibers and insufficient imaging quality. Thus, it is difficult to distinguish between boutons and non-bouton swellings using manually designed features. Further, the shapes and sizes of boutons of individual neurons in different brain regions are diverse and may include partially overlapping boutons, which renders bouton recognition difficult.

Considering these challenges, we propose here an automated method, DeepBouton, for single-neuron bouton identification in whole-brain datasets. The method includes three key parts: neuron tree division with redundancy, initial bouton detection using density-peak clustering (Rodriguez and Laio, 2014; Cheng et al., 2016), and filtering out false positives from the initial detection *via* deep convolutional neural networks (LeCun et al., 2015; He et al., 2016). DeepBouton adopts a two-step recognition strategy: density-peak clustering to detect underlying bouton centers and deep convolutional networks for filtering out non-bouton axonal swellings in the initial detection. The method combines the adaptive feature representation ability of convolutional networks and robustness of density peak clustering, allowing description of bouton morphology and segmentation of objects with various patterns including overlapping. Thus, it can effectively detect axonal swellings of various morphologies and learn high-dimensional representations of bouton morphology to distinguish reliable boutons from other candidates. In addition, we developed a neuron tree division technique to process brain-wide single neurons effectively. To validate our method, we applied it for identification of boutons of both long-range pyramidal projection neurons and local interneurons in whole-brain datasets. We obtained precision and recalls rates of approximately 0.90.

## Materials and Methods

### The Principle of DeepBouton

**Figure 1:**
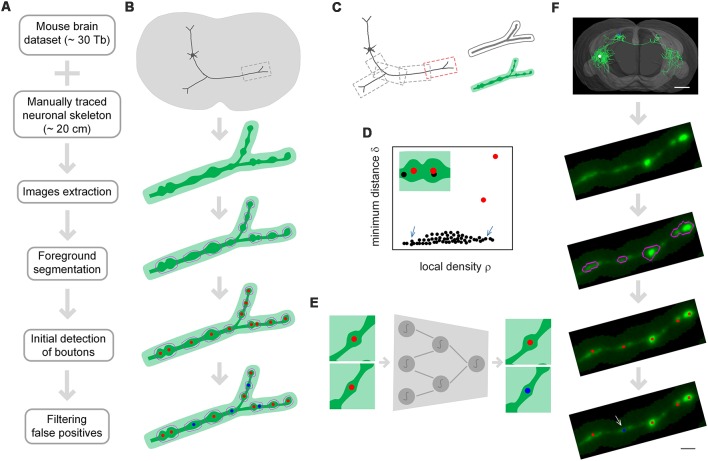
The principle of DeepBouton. **(A)** Flow diagram of DeepBouton: extract images along axons piecewise from a whole-brain dataset guided by a manually traced neuronal skeleton, segment foreground images by adaptive binarization and morphology erosion, initially detect underlying boutons using density-peak clustering, and filter non-bouton axonal swellings *via* a deep convolutional network. **(B)** Pattern graphs of DeepBouton corresponding to the flow diagram in **(A)**. **(C)** Diagram of piecewise-extracted images along axons: the axonal arbor is divided into segments with redundancy, and the tubular volume is extracted along the axonal skeleton for each segment. **(D)** Diagram of initially detected boutons using density-peak clustering: the points with a higher signal density than their neighbors and with a relatively large distance from points of higher densities are recognized as centers of underlying boutons (red dots), while the points with a higher density but with a small distance are not centers (black dots labeled by arrows). **(E)** Filtering of non-bouton axonal swellings in the initial detection *via* a patch-based classification convolutional network. **(F)** A demonstration of the method on an experimental dataset. Scale bars in **(F)** represent 1 mm and 2 μm, respectively.

DeepBouton consists of three parts: neuron tree division with redundancy, initial detection of axonal swellings, and filtering of non-bouton swellings (Figures 1A,B). First, with the guidance of a manually traced neuronal skeleton, piecewise sub-blocks are extracted along axons with redundancy (Figure 1C). For each sub-block, the foreground images are segmented through adaptive binarization and morphological erosion. Then, axonal swellings are localized with density-peak clustering in the foreground images (Figure 1D), and the detected swelling centers of all sub-blocks are merged. Finally, we designed and trained a patch-based classification convolutional network to filter the non-bouton swellings in the initial detection (Figure 1E). A demonstration of application of the method on an experimental dataset is depicted in Figure 1F.

The two-step recognition strategy is utilized for accurate identification of single-neuron boutons. The initial detection should contain as many underlying axonal swellings of diverse degrees as possible. Second recognition is then used to filter non-bouton swellings. Initial detection is difficult because underlying swellings have various sizes and partially overlap. We used density-peak clustering to locate swelling centers due to its robustness to cluster scale and effective splitting of overlapped clusters (Figure 1D). However, the initially detected swellings had diverse radii and intensities relative to neighboring axons, and a suitable recognition scale needed to be determined to distinguish boutons from non-bouton swellings. Here, we adopted deep convolutional networks to filter false positives due to their adaptive feature representation abilities without manually designed features compared to those of traditional machine learning or model-based approaches (Figure 1E). The blocking-merging strategy along axons with redundancy ensures that the method can quickly process ultra-volume datasets while maintaining recognition accuracy (Figure 1C).

### Neuron Tree Division With Redundancy

#### Image Extraction

Single-neuron boutons for long-range projection neurons generally have brain-wide distributions as axonal projections across different brain regions. Therefore, we extracted piecewise sub-block images along axonal arbors with the guidance of the reconstructed neurons. Specifically, (a) an axonal arbor is divided into several segments with redundancies; and (b) for each segment, tubular volume along the axonal skeleton with a radius of 8 × 8 × 4 voxels is extracted from the corresponding whole-brain dataset as depicted in Figure 1C. Foreground segmentation and initial detection of boutons are performed on each sub-block, and the initially detected boutons of each segment are then merged.

#### Foreground Segmentation

Foreground images are segmented through adaptive binarization and mild morphological erosion. The binarization definition is the following formulation:

$$B=\{\begin{array}{cc} 1 & I>C+thre_{\text{binarization}}\sqrt{C}\\ 0 & \text{otherwise} \end{array}$$

where *I* is the original image, *C* represents the background image generated by multiple convolutions with averaging template, and *thre*_binarization_ is a threshold parameter. It is easy to set the threshold parameter to ensure that underlying axonal bouton regions are segmented. To eliminate artifacts and noise points in binarized images, we performed mild morphological erosion. The foreground images are defined as the element-wise product of *I* and *B*.

### Initial Detection of Boutons

We located centers of axonal swellings in the segmented foreground images *via* density-peak clustering (Rodriguez and Laio, 2014; Cheng et al., 2016). The principle of density-peak clustering is searching for density peaks in the ρ, δ feature space (Figure 1D), where ρ is the local signal density (i.e., the Gaussian-weighed mean of local signal intensities), and δ is the corresponding minimum distance from voxels of higher densities. The density peaks (i.e., centers of swellings) are characterized by a higher signal density ρ than their neighbors and by a relatively large distance δ. They act as isolated points in the ρ, δ space. Therefore, possible density peaks are the voxels with low feature densities Λ defined in the ρ, δ space. The clustering method explicitly adds the minimum distance to describe cluster centers other than the local signal density. Thus, cluster centers can be searched for intuitively in the density-distance space even for multiple-scale clusters or overlapped clusters. The formulations of the density-peak clustering are provided below.

#### Formulations of Density-Peak Clustering

The local signal density ρ of each voxel is defined as follows (Cheng et al., 2016):

$$\rho_{i}=\frac{1}{Z}\sum_{j:||p_{i}-p_{j}|{|}_{2}\leq R}I(p_{j})\frac{1}{\sqrt{2\pi \sigma }}\exp\left(-\frac{||p_{i}-p_{j}|{|}_{2}^{2}}{2\sigma^{2}}\right)$$

where *I*(*p*_*i*_) represents the signal value of voxel *p*_i_; s and *R* are the kernel width and the window radius, respectively, of the Gaussian kernel function (*R* = 2σ); ||.||_2_ is the 2-norm; *Z* is a normalization constant. In our experiments, the kernel width σ is set to approximately one third of the average bouton radius.

With the density map, one can search for the minimum distance δ of each voxel according to the following formulation:

$$\delta_{i}=\{\begin{array}{cc} \frac{j:\underset{p_{j}>p_{i}}{\min}||p_{i}-p_{j}|{|}_{2}}{\underset{\forall i,j}{\max}||p_{i}-p_{j}|{|}_{2}} & \rho_{i}<\underset{\forall j}{\max}\rho_{j}\\ 1 & \rho_{i}=\underset{\forall j}{\max}\rho_{j} \end{array}$$

The density peaks (i.e., the underlying bouton centers) are characterized by a higher density ρ than their neighbors and by a relatively large distance δ. They act as isolated points in the ρ, δ space. Therefore, the possible density peaks can be selected according to the feature density Λ (the density computed in the ρ, δ space). According to this principle, we used the following formulation to search for the possible density peaks:

$$\left\{p_{j}|\text{Λ}(i)\leq thre_{\text{search}}\&\delta (i)\geq \frac{R_{\min}}{\underset{\forall i,j}{\max}|p_{i}-p_{j}{|}_{2}}\right\}$$

where *thre*_search_ is a predetermined parameter, and *R*_min_ is the minimum value of the estimated bouton radius. The setting of *thre*_search_ should ensure that the underlying bouton centers can be searched; thus, we set it to a small value.

### Filtering Out False Positives From the Initial Detection

#### The Architecture of the Deep Convolutional Network

**Figure 2:**
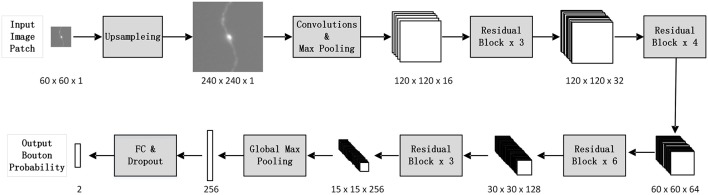
The architecture of the deep convolutional network. For each initially detected bouton center, extract its surrounding patch from the whole-brain dataset, and classify it into bouton or false bouton. The classification convolutional network is designed based on ResNet50 (He et al., 2016) according to the characteristics of bouton morphology. There are three changes compared to the original ResNet50: perform four times up-sampling of input image patches; set convolution stride as 1 and use pooling layer just once in the stem block; greatly reduce the feature map number of the original ResNet50.

We designed a patch-based classification network architecture (Figure 2) based on ResNet50 (He et al., 2016) to filter out non-bouton swellings from the initial detection according to the characteristics of bouton morphology. The biggest challenge of accurate bouton identification is how to obtain effective feature representations of bouton morphology. Since the pixel size of our dataset was 0.2 × 0.2 × 1 μm^3^, an average axonal bouton radius was about 2–3 pixels (about 0.5 μm) in the *x*-*y* plane. The scale of boutons is too small for classical classification network architecture. Thus, features of bouton morphology may disappear in sequential pooling layers of classical classification nets. We employed three strategies to address this problem: (a) performing a maximum projection of the extracted 3D image patches along the *z* axis, since the *z* resolution is insufficient to identify axonal boutons; (b) performing four-times up-sampling of the projected patches; and (c) setting convolution stride as 1 and using pooling layer just once in the stem block. Thus, the output feature map size of the stem block was half of the up-sampled image patch size instead of a quarter, as in the original ResNet50. As bouton recognition is simpler than ImageNet 1000-classification (He et al., 2016), we greatly reduced the feature map number of the original ResNet50. In addition, the dropout method (Hinton et al., 2012) and the rectified linear unit (LeCun et al., 2015) were utilized in the network to reduce the effect of overfitting and accelerate network convergence.

#### Sample Patch Preparation

The suitable patch size should contain enough neighboring neural fibers of boutons for recognition of contextual information. We set the patch size to 60 × 60 × 7 after multiple tests (i.e., volume patches with a size of 60 × 60 × 7) around the candidate bouton centers were extracted in original images. We then performed a maximum projection of the patches along the *z* axis, since the *z* resolution is insufficient to identify axonal boutons (0.2 × 0.2 × 1 μm^3^). The bouton morphological characteristics appeared more clearly in the projection image than in the 3D patch. The projection patches were up-sampled to 240 × 240 (0.05 × 0.05 μm^2^). We performed up-sampling based on two considerations: (a) axonal boutons are approximately 4–6 pixels width (about 1.0 μm) in the 60 × 60 patches, which is too small for classical classification network architecture; and (b) the learned convolutional network can adapt to different image resolutions (other resolutions can be unified to 0.05 μm).

#### Network Training

We trained the designed deep convolutional network with about 5,000 manually labeled samples (half the samples were positive). The network training was implemented through Keras (2018) with Tensorflow (2018) backend. Back propagation with mini-batch stochastic gradient decent was used during the training. A mini-batch size of 60, a learning rate of 10^−2^ with a decay of 10^−6^, and a moment of 0.9 were adopted. The network could reach the desired accuracy with approximately 50 epochs of training on one NVIDIA GTX 1080 GPU in about 1 day.

Sample augmentation is a common technique in deep learning domains of computer vision and biological image recognition. Its purpose is to add variability to the samples, thus improving the robustness, such as rotation invariance and noise immunity, of learned networks. We introduced sample augmentation in our training data as below. Rotation: rotate a sample by 90, 180, or 270 degrees. Noise: add Gaussian noise, salt and pepper noise, or Poisson noise to a sample. Shifting: shift a sample in the x-y dimension by [1, 1], [1, −1], [−1, 1], or [−1, −1]. Scaling: scale a sample by 1.2 or 0.82 rates. Transforming gray levels: multiply the image gray intensity by a random coefficient within limits.

### Ground Truth and Performance Evaluation

The criteria for manually annotating axonal boutons is that a significant axonal swelling with at least 2-fold the neighboring average axonal width is defined as a putative axonal bouton (Kalisman et al., 2005; Grillo et al., 2013). But in practice, a putative axonal bouton is judged comprehensively according to width, fluorescence intensity, morphology and neuron type. To ensure the quality and consistency of the manually annotated putative boutons of the train and test datasets, we obtained the ground truth of putative boutons by multi-expert labeling and consensus. Specifically, two PhDs majoring in neuroscience and biomedical engineering labeled putative boutons according to the above principle independently; another PhD majoring in neuroscience checked the annotations and obtained the final ground truth.

We then compared the automated recognition result with the ground truth and used precision, recall, and *F*_1_-measure to evaluate the performance of the method. Assuming *M*, *N*, and *P* are the number of the ground truth cases, automatic recognition cases, and matched cases, respectively, the precision and recall equal *P*/*N* and *P*/*M*. *F*_1_-measure is the harmonic mean of the precision and recall. If the distance between a ground truth case and an automatic case is within 1.2 μm, they are defined as a pair of matched cases.

### Materials

The experimental datasets were obtained by imaging mouse brains injected with adeno-associated virus using a high-resolution stage-scanning microscopy system (Yang et al., 2015) with chemical reactivation (Xiong et al., 2014). All experiments were performed in accordance with the guidelines of the Experimental Animal Ethics Committee at Huazhong University of Science and Technology. The protocol for sample preparation is described in detail by Gang et al. (2017). The voxel size of the imaging datasets was 0.2 × 0.2 × 1 μm^3^. Two whole-mouse brain datasets were used in this study.

In our experiment, the datasets included one training dataset and four test datasets. The training dataset contained 2,553 putative boutons as positive samples and 2,509 non-bouton axonal swellings as negative samples classified by three PhDs majoring in neuroscience and biomedical engineering. The putative boutons in test datasets were also classified by the three PhDs. The test dataset in Section “Demonstration of the Validity of DeepBouton” was comprised of 18 randomly selected sub-blocks of a pyramidal neuron. There were in total 3,831 human-classified putative boutons as the ground truth. The test dataset in Section “Applicability of DeepBouton for Multiple Neuronal Types” was comprised of seven randomly selected sub-blocks of a pyramidal neuron and a basket cell, containing in total 837 putative human-classified putative boutons as the ground truth. The test dataset in the Section “Comparisons of DeepBouton and Other Bouton Detection Methods” includes two image volumes. One is our own data with a sub axonal tree (1,501 × 1,366 × 991, 0.2 μm × 0.2 μm × 1.0 μm) containing 790 human-classified putative boutons. The other is public data with one axon trace (1,024 × 1,024 × 150, 0.26 μm × 0.26 μm × 0.8 μm) containing 35 human-classified putative boutons.

## Results

### Demonstration of the Validity of DeepBouton

**Figure 3:**
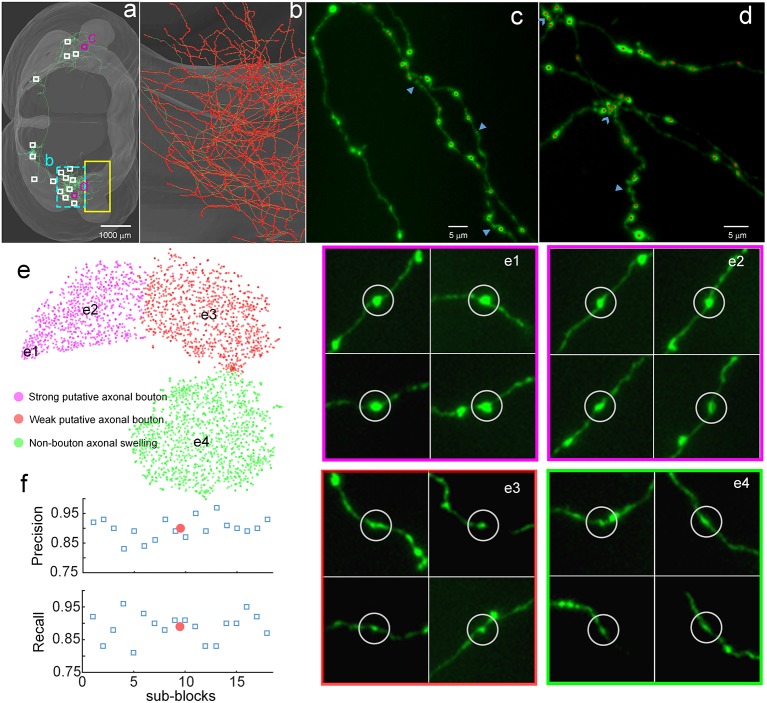
Demonstration of the validity of DeepBouton. **(A)** The traced skeleton of a pyramidal neuron in a mouse brain contour. Boutons and non-bouton swellings in the yellow-boxed region were manually labeled as training samples. A total of 18 sub-blocks were used to evaluate the recognition performance of the method. **(B)** The localization results of the method on the ipsilateral axon. The red dots are the detected bouton centers merged with the green axonal skeletons. **(C,D)** The detected boutons (red dots) in two sub-regions of the two purple-boxed evaluation regions in **(A)**, merged with the original image signal. Non-bouton axonal swellings, labeled by the triangles, were filtered out by DeepBouton. Overlapping boutons, marked by the arrows, were split. Notably, the left fiber in **(C)** does not belong to the cell; thus, its boutons were not detected. **(E)** Visualization and analysis of the learned high-dimensional features of the deep convolutional network. For each sample, we projected its high-dimensional feature representations into two dimensions using *t*-distributed Stochastic Neighbor Embedding (*t*-SNE). Colored points refer to boutons of different subtypes and non-bouton swellings. Insets of different points at several key locations are presented to aid the intuitive evaluation of the meaning of the learned features. **(F)** Recognition precision and recall rates of the 18 evaluation sub-blocks. The red points refer to average values. These figures were snapshots in Stalling et al. (2005) with 4× magnification and image contrast adjustment for better visualization.

To demonstrate the validity of DeepBouton, we applied it to identify boutons of a long-range pyramidal neuron in the motor cortex of a mouse brain dataset (Figure 3A), which was acquired by high-resolution stage-scanning microscopy with a voxel size of 0.2 × 0.2 × 1 μm^3^. The method achieved average precision and recall rates of 0.90 and 0.89 (Figure 3F) in 18 randomly selected sub-blocks (the small white-boxed or purple-boxed regions in Figure 3A), with manual annotation of a portion of boutons and non-bouton swellings of the neuron as training samples (the yellow-boxed region in Figure 3A). A total of 21,587 boutons of this pyramidal neuron were detected in the whole-brain range in approximately 4 h, including its local axon, dense ipsilateral axon (Figure 3B), and contralateral axon. Diverse non-bouton swellings (labeled by the arrows in Figures 3C,D) were filtered out by the algorithm. Overlapped boutons were split (labeled by the triangles in Figure 3D). Notably, the left fiber in Figure 3C did not belong to the cell; thus, its boutons were not detected. In conclusion, the proposed method is effective for automated identification of single-neuron boutons at the brain-wide scale.

We examined the learned high-dimensional features by visualizing them with *t*-distributed Stochastic Neighbor Embedding (*t*-SNE) dimensionality reduction (Van der Maaten and Hinton, 2008). Each point in Figure 3E represents a sample patch image projected from the 64-dimensional feature of the last hidden layer in the network into two dimensions. We observed that boutons and non-bouton swellings were separated and clustered into point clouds. In addition, two subclasses of boutons were observed and generated by *k-means* (Kanungo et al., 2002). An intuitive understanding of subclasses can be acquired by examining sample image instances: (a) strong putative boutons of larger sizes and greater intensities (insets e1 and e2 in Figure 3E), especially the boutons at the bottom left; (b) weak putative boutons of smaller sizes and lower intensities (insets e3 in Figure 3E); and (c) non-bouton swellings are derived from radius and intensity inhomogeneities of axons (insets e4 in Figure 3E). These results indicate that the features learned by the deep convolutional network represent the axonal swelling degree in radii and intensity and can be considered a comprehensive quantification of bouton morphology.

### Applicability of DeepBouton for Multiple Neuronal Types

**Figure 4:**
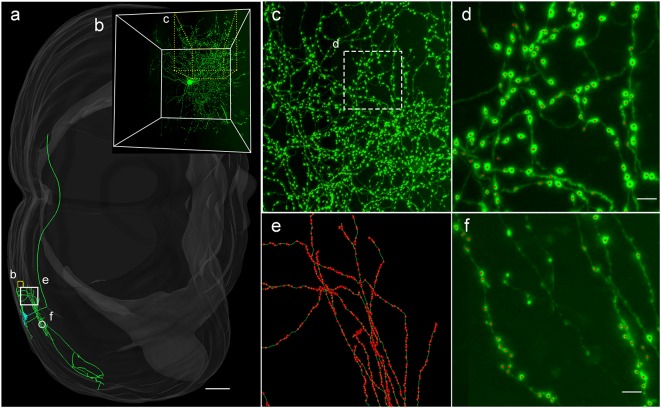
Applicability of DeepBouton for multiple neuronal types. **(A)** The traced skeleton of a pyramidal neuron in a mouse brain contour. **(B)** The original image signal of a basket cell in the same brain region as the pyramidal neuron. **(C,D)** The automatic bouton detection results labeled by red dots of the basket cell. Dots in **(C)** were too small; thus, we enlarged a subregion of **(D)**. **(E,F)** The recognition results of the pyramidal neuron in a big branch and local small region. Scale bars represent 500 μm in **(A)** and 3 μm in **(D,F)**. Notably, the middle fiber in **(F)** does not belong to the cell; thus, its boutons were not detected. The image contrast of these figures was adjusted for better visualization.

To demonstrate the wide applicability of DeepBouton, we applied it to additional types of neurons, including an interneuron (basket cell) and pyramidal neuron in the primary somatosensory barrel cortex of another mouse brain dataset. The interneuron was locally distributed, and the pyramidal neuron was a long-range projection neuron (Figures 4A,B). The method achieved 0.97 and 0.92 average *F*_1_-measures for the two neurons, respectively; although their bouton morphology and distributions were different. Boutons of the basket cell were strong and large but densely distributed, while boutons of the pyramidal cell had diverse sizes and intensities and were widely distributed. For both cells, DeepBouton obtained effective recognition results (Figures 4C–F). Notably, the middle fiber in Figure 4F did not belong to the cell; thus, its boutons were not detected. Detailed performance statistics are provided in Table 1.

**Table 1:** Performance of single-bouton recognition using DeepBouton.

Interneuron	Precision	Recall	*F*_1_-measure	Pyramidal neuron	Precision	Recall	*F*_1_-measure
Block 1	0.96	0.99	0.98	Block 1	0.97	0.93	0.95
Block 2	0.95	0.98	0.96	Block 2	0.98	0.91	0.95
Mean ± S.D	0.96 ± 0.007	0.98 ± 0.006	0.97 ± 0.007	Block 3	0.95	0.81	0.88
				Block 4	0.96	0.89	0.93
				Block 5	0.89	0.89	0.89
				Mean ± S.D	0.95 ± 0.03	0.89 ± 0.04	0.92 ± 0.03

### Comparisons of DeepBouton and Other Bouton Detection Methods

**Figure 5:**
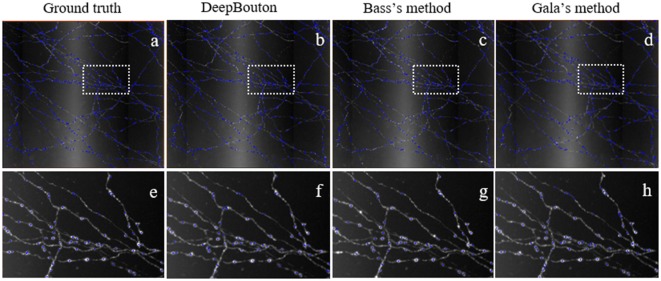
Comparisons of DeepBouton and other bouton detection methods in our own dataset.**(A)** The ground truth boutons in the dataset. **(B,C,D)** The detected boutons by DeepBouton, Bass’s method and Gala’s method in the dataset separately. **(E,F,G,H)** The enlarged view of the same sub-region in **(A,B,C,D)**.

**Figure 6:**
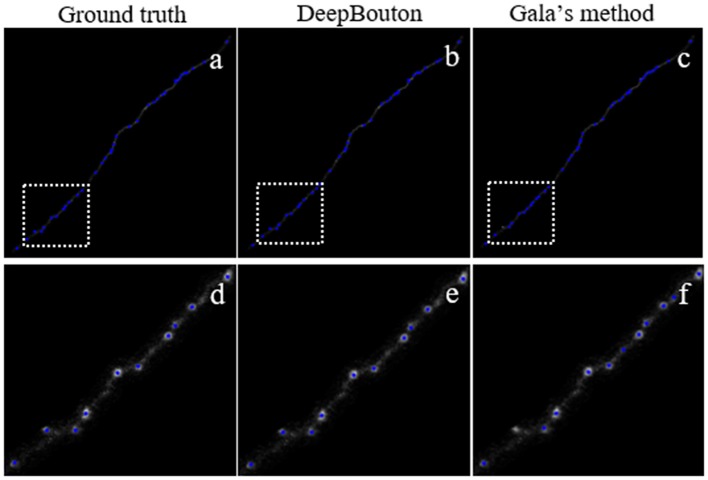
Comparisons of DeepBouton and other bouton detection methods in a public dataset.**(A)** The ground truth boutons in the public dataset. **(B,C)** The detected boutons by DeepBouton and Gala’s method in the public dataset separately. **(D,E,F)** The enlarged view of the same sub-region in **(A,B,C)**.

To further demonstrate the validation of our method, we compared DeepBouton and other bouton detection methods on two datasets including our own dataset and a public dataset. Gala’s method (Gala et al., 2017) detects boutons through finding peaks in the intensity profile along axonal traces and gives the weight of each detected bouton. Bass’s method (Drawitsch et al., 2018) detects bouton-based Gabor or HOG features and support vector machine to classify axonal swellings. Our own dataset is a 1,501 × 1,366 × 991 (0.2 μm × 0.2 μm × 1.0 μm) volume image containing a sub tree of a pyramidal neuron. There are 790 manually annotated putative axonal boutons in the sub tree. The public dataset is 1,024 × 1,024 × 150 (0.26 μm × 0.26 μm × 0.8 μm) with one axon trace from Gala et al. (2017)^3^. However, no manually annotated boutons were provided accompanying the dataset. Thus, we labeled the boutons of the dataset by two experts and obtained the ground truth by consensus. There were 35 putative boutons on the axon trace. Our method achieved higher precision and recall compared with Gala’s method and Bass’s method (Table 2). Figures 5, 6 show the detection boutons of different methods. Notably, we used a bouton weight threshold of 1.4 in Gala’s method, which was a value to achieve the best F1-measure. The image resolution parameter was adjusted for different datasets. When applying DeepBouton to the public dataset, we did not adjust the parameters and retrained the convolutional network. The Bass’s method did not get reasonable results on the public dataset, therefore we did not compute its precision and recall in Table 2.

**Table 2:** Comparison of DeepBouton and other bouton detection methods.

		Detected bouton number	Precision	Recall	*F*1-measure
Dataset1	DeepBouton	750	0.95	0.91	0.93
	Bass’s method	626	0.93	0.74	0.82
	Gala’s method	804	0.82	0.84	0.83
Dataset2	DeepBouton	36	0.92	0.94	0.93
	Bass’s method	N/A	N/A	N/A	N/A
	Gala’s method	39	0.85	0.94	0.89

### The Role of the Deep Convolutional Network

**Figure 7:**
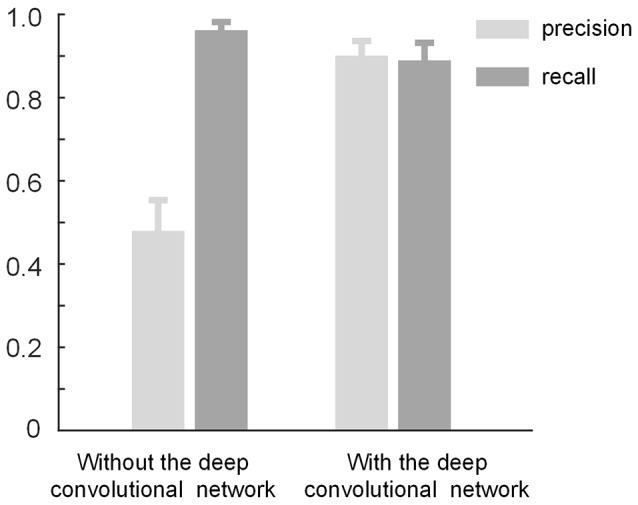
The role of the deep convolutional network. The initial bouton detection using density-peak clustering without the convolutional network achieves a high recall but low precision of ≈ 0.5. The precision can be improved to ≈ 0.9 by adding the convolutional network to filter out the non-bouton swellings in the initial detection while mildly reducing the recall.

The proposed method consists of two parts: density-peak clustering for initial bouton detection and the deep convolutional network for filtering out non-bouton swellings from the initial detection. Here, we demonstrate the effects of the two parts. We used the method with and without the convolutional network to test the dataset in Figure 3. Based on the performance of the two methods (Figure 7), it is clear that: (a) the method without the convolutional network achieves a high recall but low precision (i.e., the initial detection includes underlying boutons but also contains approximately 50% false positives); (b) the precision can be improved to approximately 0.9 by adding the convolutional network to filter out the false positives while only minimally reducing the recall. The results are consistent with the intentions of our two-step recognition strategy.

## Discussion

Identification and quantitative analysis of single-neuron axonal boutons in their entirety is critical for understanding the wiring patterns of neural circuits. However, limited methods are available for the automated identification of single-neuron boutons, even though sparse-labeling fine-imaging whole-brain datasets have been obtained. In this article, we proposed an automated recognition method, DeepBouton, based on density-peak clustering and deep convolutional networks. We demonstrated its validity in detecting single-neuron axonal boutons at the brain-wide scale and its applicability for multiple types of neurons.

Synaptic connectivity inference is a key step for mapping neural circuits (Lichtman and Denk, 2011; Helmstaedter and Mitra, 2012). Currently, electron microscopy and fluorescence optical microscopy are the two main tools for imaging brain circuits (Helmstaedter and Mitra, 2012; Osten and Margrie, 2013). Synapses can be identified in electron microscopic images, and many automated detection algorithms based on models or learning have been developed (Kreshuk et al., 2011; Dorkenwald et al., 2017). However, electron microscopy is a small volume imaging technique, and it is almost impossible to acquire single-neuron synapses at a brain-wide scale. Most axonal synapses (i.e., presynaptic structures), appear as axonal boutons in light microscopy (Hellwig et al., 1994; Anderson et al., 1998). Recent reports by Drawitsch et al. (2018) and Gala et al. (2017) demonstrated that light microscopy-based axonal boutons were highly correlative with electron microscopy. Though axonal synapses cannot be confirmed in light microscopic images, axonal boutons identified by DeepBouton reflect axonal synapse distribution in statistical terms. Therefore, we can infer synaptic connectivity at the single-neuron level by counting axonal boutons in whole-brain light microscopic images using DeepBouton.

**Figure 8:**
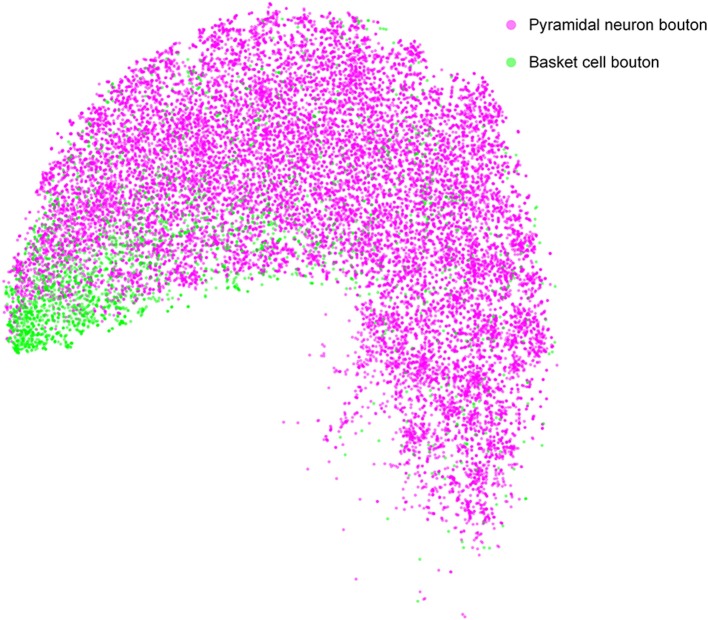
Comparison of bouton distribution of pyramidal neurons and basket neurons in the learned feature space of DeepBouton. Purple and green points refer to the detected boutons of pyramidal neurons and basket cells, respectively. The boutons of the former cell type are distributed uniformly, while those of the latter cell type are clustered at the bottom left in the projection feature space.

Many quantitative analyses of single-neuron bouton distribution patterns will be facilitated by using DeepBouton. As an example, we compared the bouton distributions between pyramidal cells and basket cells in the feature representation space to analyze the differences in their bouton morphologies (Figure 8). The results indicate that boutons of basket cells are strong and larger, corresponding to strong putative boutons in Figure 3E; while boutons of pyramidal neurons are of diverse sizes and intensities (i.e., strong putative boutons and weak putative boutons were distributed uniformly).

We released the code of DeepBouton with two test datasets and provided a user manual^4^. The section of the initial bouton detection is implemented in Matlab and the section of the convolutional network is implemented in Python. Few parameters of the initial detection need to be changed for new datasets. However, the convolution network trained on our own datasets may need to be refined if the new datasets are very different from our dataset.

In the future, a software platform based on this method will be developed for accurate and automated identification of single-neuron boutons at the brain-wide scale and to perform rapid manual verification of the automated detection. Using the platform, more detailed quantitative analyses of the distribution of various subtypes of single-neuron boutons in different brain regions will be possible.

## Author Contributions

SZ and HG conceived the project. SZ, SC, XW, and LS designed the model. SC developed the algorithm. YL, XW, NL, FY, HG and QL acquired the fluorescence datasets. SC, TQ, FX and XL performed the image analysis and processing. YL, SC and XW obtained the ground truth. SC, XW and SZ wrote the manuscript. All the authors revised the manuscript.

## Conflict of Interest Statement

The authors declare that the research was conducted in the absence of any commercial or financial relationships that could be construed as a potential conflict of interest.

## Footnotes

^1^http://www.northeastern.edu/neurogeometry/resources/bouton-analyzer/

^2^https://github.com/ShenghuaCheng/DeepBouton
